# A Proteomic Approach to Understand the Role of the Outer Membrane Porins in the Organic Solvent-Tolerance of *Pseudomonas aeruginosa* PseA

**DOI:** 10.1371/journal.pone.0103788

**Published:** 2014-08-04

**Authors:** R. Hemamalini, Sunil Khare

**Affiliations:** Enzyme and Microbial Biochemistry Lab, Department of Chemistry, Indian Institute of Technology, Delhi, New Delhi, India; Louisiana State University and A & M College, United States of America

## Abstract

Solvent-tolerant microbes have the unique ability to thrive in presence of organic solvents. The present study describes the effect of increasing hydrophobicity (log P_ow_ values) of organic solvents on the outer membrane proteome of the solvent-tolerant *Pseudomonas aeruginosa* PseA cells. The cells were grown in a medium containing 33% (v/v) alkanes of increasing log P_ow_ values. The outer membrane proteins were extracted by alkaline extraction from the late log phase cells and changes in the protein expression were studied by 2-D gel electrophoresis. Seven protein spots showed significant differential expression in the solvent exposed cells. The tryptic digest of the differentially regulated proteins were identified by LC-ESI MS/MS. The identity of these proteins matched with porins OprD, OprE, OprF, OprH, Opr86, LPS assembly protein and A-type flagellin. The reported pI values of these proteins were in the range of 4.94–8.67 and the molecular weights were in the range of 19.5–104.5 kDa. The results suggest significant down-regulation of the A-type flagellin, OprF and OprD and up-regulation of OprE, OprH, Opr86 and LPS assembly protein in presence of organic solvents. OprF and OprD are implicated in antibiotic uptake and outer membrane stability, whereas A-type flagellin confers motility and chemotaxis. Up-regulated OprE is an anaerobically-induced porin while Opr86 is responsible for transport of small molecules and assembly of the outer membrane proteins. Differential regulation of the above porins clearly indicates their role in adaptation to solvent exposure.

## Introduction

Organic solvents have been in use as disinfectants and microbicidal agents for a long time. However, the discovery of solvent-tolerant *Pseudomonas putida* by Inoue and Horikoshi [1] initiated understanding of a class of microorganisms described as ‘solvent-tolerant microorganisms’ capable of growing at high concentrations of organic solvents [1], [2]. For instance, strains of *Pseudomonas putida* tolerant to toluene, *Clostridium acetobutylicum* to butanol, *Escherichia coli* to ethanol are known among the Gram-negative bacteria [3]. On the other hand, *Arthrobacter* and *Flavobacterium* resistant to benzene, *Bacillus* resistant to chloroform, n-butanol and benzene and benzene-tolerant *Rhodococcus* strains are known from Gram-positive bacteria [4], [5].

There is an increasing interest in such organisms due to their effectiveness in solvent bioremediation and biotransformations in non-aqueous media [2]. The unique properties of these microbes also kindled interest in understanding and elucidating their adaptation mechanism. In general, microorganisms adapt to organic solvents by (i) partitioning the solvent in the lipid layer, (ii) isomerisation of *cis-* unsaturated fatty acids to *trans*- unsaturated fatty acids leading to denser membranes, (iii) changing the saturated-to-unsaturated fatty acid ratio (iv) changes in length of the acyl-chains and (v) changes in the phospholipid head groups [6]. Since the outer membrane proteins (OMPs) are the primary targets of solvents surrounding the cells, the outer membrane sub-proteome would be a sensitive response indicator for studying the effect of solvents [7].

So far, proteomics studies on the bacteria exposed to solvents have been focused on highly toxic solvents such as aliphatic and aromatic alcohols, and alkyl benzenes. For instance, the effect of phenol on the membrane proteome of *Pseudomonas*, have shown an increase in the expression levels of the solvent efflux pump systems (e.g. TtgA, TtgC, Ttg2A, Ttg2C, PP_1516-7) and a decreased content of porins OprB, OprF, OprG and OprQ [8]. An imp/ostA encoded 87 kDa, outer membrane protein has been implicated in n-hexane tolerance in *Escherichi coli* [9]. TolC, the outer membrane component of the AcrAB-TolC solvent efflux pump found in *Escherichia coli* is suggested to play a crucial role in imparting solvent tolerance [10]. While a substantial understanding of the mechanisms underlying the adaptation of solvent-tolerant bacteria towards aromatic compounds has emerged, the effect of alkanes has not been investigated systematically.

Alkanes are common environmental effluents as they constitute 20–50% of crude oil [11]. They occur as mixtures in crude oil and are also among the commonly encountered industrial solvents, yet systematic studies on their uptake by microbial cells, membrane adaptations, change in protein expression and proteomic profiles have not been much worked out. Hence, it seems quite worthwhile to investigate the interaction of alkanes with cells and understand the cellular response.

The present work aims at evaluating the effect of alkanes of increasing hydrophobicity (log P_ow_) such as n-hexane, cyclohexane, n-heptane, n-octane, n-decane, n-dodecane and n-tetradecane on the proteome profile of a solvent-tolerant *Pseudomonas* strain, especially to see which proteins get differentially expressed in response to solvent exposure and thus play a role in the cellular adaptation. Since the outer membrane is the first to interface the outer environment, the focus of the present study is the outer membrane proteome. A well characterized solvent-tolerant *Pseudomonas aeruginosa* strain PseA (MTCC 10634), previously isolated by us has been used as a model system [12]–[14]. The cells grown in the medium containing high amount of alkanes were studied for the changes in the outer membrane proteome using two-dimensional gel electrophoresis followed by identification of the differentially expressed proteins by ESI-Mass. The results indicate that the outer membrane porins show significant differential expression in presence of alkanes. The differential expression of type A flagellin, in response to various alkanes is observed first time, although its involvement in toluene tolerance has been shown previously [15].

## Methods

### Chemicals

Yeast extract and peptone were purchased from HiMedia Laboratories (Mumbai, India). Acrylamide, bisacrylamide, iodoacetamide IPG strips, IPG buffer and DryStrip cover fluid were obtained from GE Healtcare (Uppsala, Sweden), DTT and DeStreak Reagent were from PlusOne (Germany). Formic acid was procured from Merck (Darmstadt, Germany). Molecular weight markers were from Bangalore Genei (Bangalore, India). In-gel tryptic digestion kit was from Sigma-Aldrich (St. Louis, USA). All other chemicals used were of analytical grade.

### Bacterial strain and growth conditions

*Pseudomonas aeruginosa* strain PseA, (MTCC 10634), an organic solvent-tolerant microorganism was maintained and subcultured as described previously [12]. Inoculum was prepared by transferring a loopful of this stock culture to the growth medium prepared in nutrient broth (NB) containing (g/L) yeast extract 3.0, peptone 5.0 and NaCl 0.5 The pH was adjusted to 7.2 and the culture was grown at 30°C with shaking at 120 rpm until the absorbance at 660 nm (A_660_) reached 1.0. To check the effect of organic solvents on the proteome of *P. aeruginosa*, 2% (v/v) of the inoculum was used to seed eight identical 500 ml Erlenmeyer flasks containing 100 ml of growth medium which was then overlaid with 50 ml of the solvent. The flasks were sealed with butyl rubber stoppers to prevent the evaporation of solvents and incubated at 30°C and 140 rpm until late log phase was reached. *P. aeruginosa* was also grown in NB, in absence of solvent as a control under similar conditions. Growth was monitored by recording absorbance at 660 nm. The aqueous phase, containing the cells, was carefully pipetted out from beneath the organic layer and the cells were harvested by centrifugation for 15 minutes at 10,000×*g* at 4°C. The cells were washed with ice cold Tris-HCl buffer (0.02 M, pH 8.5) and the pellet was stored at −80°C.

### Extraction of bacterial membrane proteins

The cell pellets were processed for membrane proteome sample preparation following a modified form of the method described previously [8]. Briefly, 3 stored cell pellets each from control and solvent treated cultures were resuspended in chilled lysis buffer (0.020 M Tris HCl, pH 8.5) containing 25 mM NaCl, 2 mM EDTA, 10% (v/v) glycerol, 0.5% (v/v) Triton X-100, 10 mM 2-mercaptoethanol, 1 mM DTT, 1 mM PMSF, 10 mM NaF. The suspension was sonicated twice on ice using a cycle of 40%, for 5 min, 120 watts at a frequency of 20 kHz on a Biologics Ultrasonic Homogenizer (Virginia, USA). The sonicated suspension was centrifuged for 30 min at 10,000×*g* at 4°C. The pellets were discarded. The supernatant was diluted with ice cold sodium carbonate solution (0.1 M, pH 11.0) and stirred slowly on ice for 1 h [14]. The carbonate treated membrane fraction was ultracentrifuged in a Beckman 70 Ti rotor for 1 h at 99,000×*g* at 4°C. The pellet was suspended in 16 ml of 50 mM Tris HCl, pH 7.2 and the ultracentrifugation step repeated. The pellets obtained at the end consisted of soluble membrane proteins. They were dissolved in DeStreak reagent (Plus One, GE Healthcare) and processed further.

### 2-D Electrophoresis

Proteins present in the total membrane fraction were quantified by Bradford assay [15]. Overnight passive rehydration of 13 cm, 3–10 NL IPG strips (GE Healthcare) were carried out in presence of 250 µl of sample containing 300 µg of protein and 2% (v/v) IPG buffer containing pharmalytes for the 3–10 NL IPG strips. Isoelectric focusing was carried out on an Ettan IPGphor 3 instrument (GE Healthcare, Buckinghamshire, UK) for 15,000 Vh at a maximum voltage of 8000 V. After the IEF run, the IPG strips were incubated in the SDS equilibration buffer (6 M Urea, 75 mM Tris-HCl pH 8.8, 29.3% Glycerol, 2% SDS, 0.002% bromophenol blue) containing DTT 1% (w/v) for 15 min followed by incubation in the same buffer containing iodoacetamide 2.5% (w/v) for 15 min. Second dimension electrophoresis was carried out on SE 600 Ruby (GE Healthcare) by placing the equilibrated IPG strips horizontally over 1.5 mm thick, 12.5% SDS-polyacrylamide gels and sealed in place with molten agarose sealing solution (25 mM Tris base, 192 mM glycine, 0.1% SDS, 0.5% agarose, 0.002% bromophenol blue). Electrophoresis was carried out in two steps: first step at 15 mA/gel for 15 min followed by the second step at 50 mA/gel till the dye front was 1 mm from the bottom of the gel. Gels were stained with Coomassie Brilliant Blue R-250 and then destained in a solution of 30% (v/v) methanol and 10% (v/v) acetic acid. All the gels were scanned on a BioRad Gel doc system, Universal Hood II and the images were saved for further spot analysis.

### Analysis of protein expression levels

Gel images were analyzed using Image Master Platinum 7.0 software (GE Healthcare). Protein spots were identified by using the automatic spot detection algorithm. Individual spot volumes were normalized against total spot volumes for a given gel. Statistical analysis was also performed by the Image Master Platinum 7.0 software package. Averages of protein abundance for membrane fraction of each growth condition were also compared by their normalized volume using one-way ANOVA between test groups. This test returned a p-value that takes into account the mean difference and the variance of a matched spot between sample conditions and also the sample size. Only statistically significant spots (p<0.05) were selected for analysis. Differential expression between the different solvents and control was quantified on the basis of volume percent. Spots that showed at least 1.5 fold change in their expression levels and clearly visible on the 2D gel were considered for further analysis. Protein spots that were absent from control or in all the solvent-treated samples, were also considered for analysis. Triplicate gels were run for control and each of the seven solvent treated samples.

### Identification of proteins by liquid chromatography-electrospray ionization-tandem mass spectrometry (LC-ESI-MS/MS)

Protein spots that showed significant differential expression were excised and subjected to tryptic in-gel digestion using the Sigma-Aldrich in-gel tryptic digestion kit (Sigma-Aldrich, St. Louis, USA) as per manufacturer’s instruction. The digests were kept at −20°C prior to analysis and then eluted on a 2.2 µm, 120°A (2.1×150 mm) Dionex column (Acclaim TM RSLC C18) using LC (Dionex Ultimate 3000) (Thermo Scientific, Geel, Belgium). Mobile phase A was composed of 0.1% (v/v) formic acid in water. Mobile phase B was 0.1% (v/v) formic acid in ACN. 40 µl of sample was injected and the percentage of mobile phase B was increased linearly from 10 to 75% (v/v) in 25 min and from 75 to 90% (v/v) in 10 min, maintained at 90% (v/v) for 10 more min before finally reducing to 10% (v/v) in 10 min. The column effluent was connected to an ESI nano-sprayer on microTOF-QII (Bruker, Bremen, Germany). In the survey scan, MS spectra were acquired for 1 min in the m/z range between 400 and 1400. The mass data was analyzed on the Compass Data Analysis software and a mascot generic file format was generated which was then used for automated protein identification through a library search using the Mascot 2.4.01 search engine (Matrix Science, London, UK) through the BioTools software version 3.0 (Bruker Daltonics, Billerica, USA). In the MS/MS Ion Search dialog box, the taxonomy was set to a ‘All entries’, NCBInr or SwissProt databases were used for the different searches, enzyme trypsin was selected from the list, partial (cleavages per peptide) was set to 1, global modification was set to carbamidomethyl (C) and the variable modification was set to Oxidation (M). The mono-isotopic peptide mass tolerance was set to 25 to 75 ppm depending on the sample while its fragment ion mass tolerance was set to 0.2 Da. Mascot scores above the zone of significance were taken to be valid for the protein identity.

### Determination of protein hydrophobicity

The hydrophobicity of a protein is an important property of membrane proteins. In the present study, the theoretical hydrophobicity of the identified proteins was determined by an *in-silico* approach. The grand average hydropathy (GRAVY) value is a measure of the hydrophobicity of a protein [16]. For each of the identified proteins, the aminoacid sequence in the FASTA format was submitted to the online GRAVY calculator (www.gravy-calculator.de) in order to determine the GRAVY score. This value was then used to correlate the hydrophobic nature of the protein and its expression pattern in response to the hydrophobic solvent stress.

## Results and Discussion

### Effect of solvents on the growth

Solvent-tolerant Gram-negative bacteria adapt by making certain changes in outer membrane composition and structure in response to solvent exposure [3]. In the present study, the effect of alkanes was analyzed on a known solvent-tolerant *Pseudomonas aeruginosa* strain. The strain was grown in NB media containing 33% (v/v) alkanes of varying hydrophobicity and its outer membrane proteome was analyzed to elucidate the up-regulation and down-regulation of specific proteins involved in membrane-level response to the increasing hydrophobicity of solvent. The control consisted of cells grown in NB media without addition of any organic solvent.

**Figure 1:**
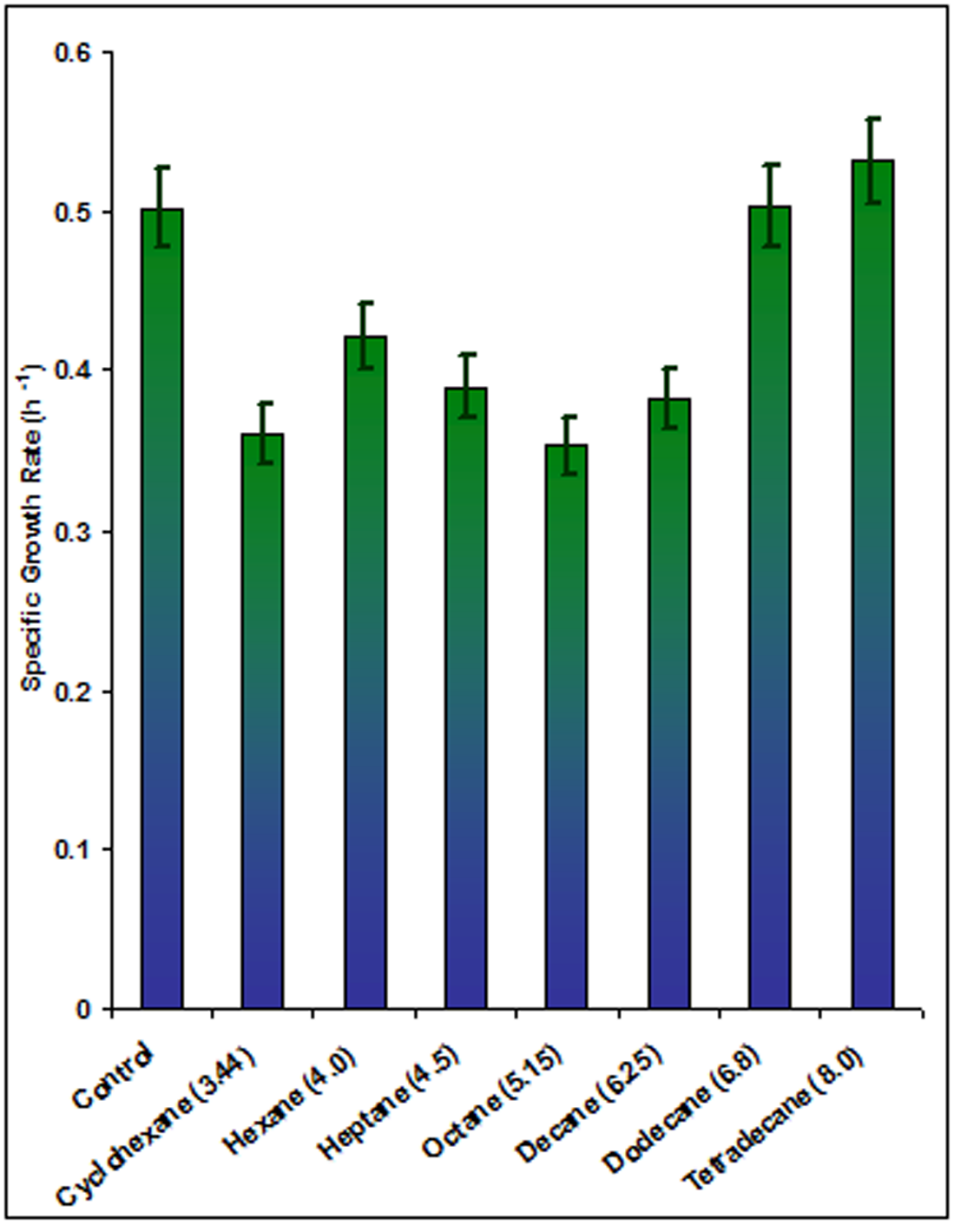
Effect of solvent polarity on the specific growth rate of *Pseudomonas aeruginosa* PseA. The log P_ow_ values have been indicated in parentheses.

The toxicity of a solvent and its effect on growth is generally monitored with respect to the hydrophobicity of that solvent, which is represented as the hydrophobicity index called log P_ow_ value (defined as the partition coefficient of a solvent in an equimolar mixture of octanol and water). Solvents with lower log P_ow_ values have been reported to be more toxic to living cells [17]. The effect of solvents of varying log P_ow_ on the growth of the solvent-tolerant *Pseudomonas aeruginosa* PseA is shown in Figure 1. The specific growth rate (µ) was reduced by 16–30% in presence of solvents cyclohexane, n-hexane, n-heptane, n-octane and n-decane (log P_ow_ 3.44–6.25), cyclohexane causing maximum reduction. The growth remained unaffected in presence of solvents of higher hydrophobicity index, n-dodecane (log P_ow_ 6.80) and n-tetradecane (log P_ow_ 8.00). Evidently, solvents with a lower log P_ow_ value affect growth more adversely.

The *Pseudomonas* strain used herein was previously isolated by cyclohexane enrichment from soil samples in the vicinity of a solvent extraction unit in New Delhi, India [12]. It has been well characterized for its solvent-tolerant traits [2], [12], [13]. A previous study on the cellular mechanisms for solvent tolerance of this strain revealed that cyclohexane (log P_ow_) altered the cell growth, morphology, size, membrane integrity, permeability and surface hydrophobicity, whereas n-tetradecane (log P_ow_) did not affect any of these parameters [18].

Loss of membrane integrity is reported to cause the leakage of ATP, ions, nucleic acids, phospholipids and proteins [3]. Similar leakage of dihydrolipoamide dehydrogenase, GroEL and other cytoplasmic proteins also occurred into the secretome of *Pseudomonas aeruginosa* PseA cells treated with solvents, n-hexane, cyclohexane, n-heptane and n-octane (data not shown). This observation further supports damaged/leaky membrane in presence of solvents of low log P_ow_ values. The reduction in the specific growth rate of the bacterial strain in presence of cyclohexane to n-decane can therefore be explained in the light of the more toxic effect of these solvents on the membrane, as compared with higher log P_ow_ solvents.

### Effects of solvents on the outer membrane of *Pseudomonas aeruginosa*

**Figure 2:**
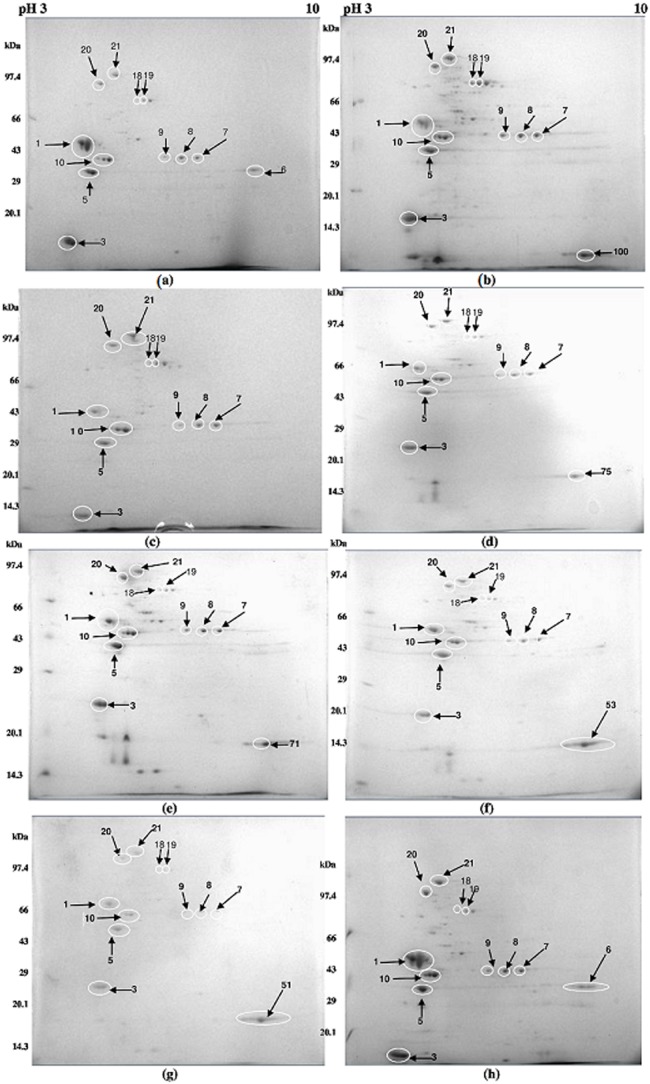
Outer membrane proteome of *Pseudomonas aeruginosa* PseA grown in presence of various alkanes. The *Pseudomonas aeruginosa* cells were grown in NB medium containing 33% (v/v) solvents, pH 7.2, at 30°C and 120 rpm. The cells were harvested in late log phase and their outer membrane proteins extracted following alkaline extraction and ultracentrifugation. The proteins so obtained were then subjected to 2D electrophoresis. The results are of cells grown in (a) control, only NB medium. (b) NB medium + n-hexane (c) NB medium + cyclohexane (d) NB medium + n-heptane (e) NB medium + n-octane (f) NB medium + n-decane (g) NB medium + n-dodecane (h) NB medium + n-tetradecane.

Exposure to phenol has been shown earlier to lead to a coordinate increase in solvent efflux pump proteins such as TtgA, TtgC, Ttg2A, Ttg2C, and PP_1516-7 and a decreased content of porins OprB, OprF, OprG and OprQ [8]. Considering the fact that outer membrane is the first to be affected, the control and solvent-grown cells were harvested in the late log phase and processed for isolating the outer membrane proteins. The outer membrane proteins from each sample were subjected to 2-D gel electrophoresis in triplicates. The gels were scanned using BioRad Gel doc system. The outer membrane proteome map for control and each of the solvent-treated samples are shown in Figure 2 (a–h). The proteome spots were analyzed by Image Master Platinum 7.0 software. A total of 117 statistically significant spots (threshold p-value<0.05) were detected in the control and 7 gels. Number of proteins detected on the 2-D gels varied across the different solvent-treated samples. All these spots were seen to have a pI between 3–8 and molecular mass in the range of 14–40 kDa, as suggested by their position in the 2-D gel.

Further analysis by Image Master revealed that twelve spots were significantly, differentially expressed. The results are summarized in Table 1. Among these proteins spot numbers 1, 3, 5, 10 and 18 were down-regulated while 7, 8, 9, 19, 20 and 21 were up-regulated. Most of the spots show significant changes in case of cyclohexane-treated cells.

**Table 1:** Differential expression of outer membrane proteins in *Pseudomonas aeruginosa* grown in presence of alkanes.

Protein spot no.	Effect on expression levels in the solvent treated cells*						
	n-Hexane	Cyclohexane	n-Heptane	n-Octane	n-Decane	n-Dodecane	n-Tetradecane
1		↓				↓	
3					↓		
5	↓	↓			↓	↓	↓
6							↓
7		↑	↑		↑	↑	
8		↑					
9	↑	↑	↑				
10	↓	↓	↓	↓	↓	↓	
18				↓	↓		
19		↑					
20		↑	↑			↑	
21		↑	↑			↑	

*Protein spots that are seen in control as well as solvent-treated cells but showing different level of expression.

↓Solvent-treated cells showing significant reduction in expression levels.

↑Solvent-treated cells showing significant increase in expression levels.

Protein spot 1 was down-regulated to the maximum extent in case of cyclohexane and n-dodecane exposed cells. The spot 10 showed a considerable variation in expression, mostly down-regulated in all the solvents, except in case of n-tetradecane. Protein spot 6 of the control was seen only in n-tetradecane with a significant down-regulation. This spot disappeared in all other solvents.

Interestingly, some spots which were not seen in control appeared as altogether new spots in response to solvents. These were 51 for dodecane, 53 for decane, 71 for octane, 75 for heptane and 100 for n-hexane. These spots were all significantly up-regulated and were analysed for determining their identity.

### Characterization and functional correlation of differentially expressed proteins

Protein spots showing significant differential expression were excised and subjected to in-gel tryptic digestion and subjected to LC-ESI-MS/MS analysis of tryptic digests. The results were matched with databases and identified using the Mascot search engine. Only matches in the significant region and those with the top most score were considered. The identity, characteristics and possible functions of the differentially expressed proteins are summarized in Table 2. The theoretical pI values of these proteins, as per the database, were in the range of 4.94–8.67 and the molecular weights ranged between 19.5–104.5 kDa. All these proteins were localized to the outer membrane.

**Table 2:** Characteristics and function of outer membrane proteins of *Pseudomonas aeruginosa* PseA grown in alkanes.

Protein spot no.	Gene	Protein name	NCBI Accession number	Molecular weight kDa	pI	Mascot score	Number of matched peptides	Sequence coverage (%)	GRAVY	Function
**1**	oprD	Outer membrane porinOprD family porin	gi|386066727	46.9	5.45	791	12	29	−0.43	Allow entry of aminoacids, peptides and carbapenems into cell [25]
**3**	oprF	Outer membrane porin F	gi|15596974	37.8	4.98	202	4	19	−0.44	Involved in cell structure maintenance, outer membrane permeability, environment and host immune system sensing, and virulence [33]–[36]
**5**	oprF	Outer membrane porin F	gi|15596974	37.8	4.98	202	4	19	−0.44	Involved in cell structure maintenance, outer membrane permeability, environment and host immune system sensing, and virulence [33]–[36]
**6**	oprH	Chain A, Solution Structure Of Outer Membrane Protein H (OprH) In Dhpc Micelles	gi|344189438	19.5	8.11	1145	18	73	−0.63	Prevent uptake of antimicrobials [37]
**7**	oprE	Anaerobically-induced outer membrane porin OprE precursor	gi|15595488	49.6	8.67	264	4	9	−0.44	Involved in transport of small molecules and is anaerobically induced [38]
**8**	oprE	Anaerobically-induced outer membrane porin OprE precursor	gi|15595488	49.6	8.67	264	4	9	−0.44	Involved in transport of small molecules and is anaerobically induced [38]
**9**	oprE	Anaerobically-induced outer membrane porin OprE precursor	gi|15595488	49.6	8.67	264	4	9	−0.44	Involved in transport of small molecules and is anaerobically induced [38]
**10**	fliC	A-type flagellin	gi|126165550	40.0	4.94	904	12	37	−0.13	Structural units of flagella [39]
**20**	opr86	Outer membrane porin Opr86	gi|15598844	88.3	5.05	1197	15	30	−0.37	Responsible for outer membrane protein assembly, cell viability and biofilm formation [27]
**21**	lptD	LPS assembly protein	gi| 25008883	104.6	5.37	314	4	5	−0.6	Role in lipopolysaccharide assembly in the outer membrane [28]
**51**	oprH	Chain A, Solution Structure Of Outer Membrane Protein H (Oprh) InDhpc Micelles	gi|344189438	19.5	8.11	1145	18	73	−0.63	Prevent uptake of antimicrobials [37]
**53**	oprH	Chain A, Solution Structure Of Outer Membrane Protein H (Oprh) In Dhpc Micelles	gi|344189438	19.5	8.11	1145	18	73	−0.63	Prevent uptake of antimicrobials [37]
**71**	oprH	Chain A, Solution Structure Of Outer Membrane Protein H (Oprh) In Dhpc Micelles	gi|344189438	19.5	8.11	1145	18	73	−0.63	Prevent uptake of antimicrobials [37]
**75**	oprH	Chain A, Solution Structure Of Outer Membrane Protein H (Oprh) In Dhpc Micelles	gi|344189438	19.5	8.11	1145	18	73	−0.63	Prevent uptake of antimicrobials [37]
**100**	oprH	Chain A, Solution Structure Of Outer Membrane Protein H (OprH) In Dhpc Micelles	gi|344189438	19.5	8.11	1145	18	73	−0.63	Prevent uptake of antimicrobials [37]

Protein spot 1 matched with OprD, 3 and 5 with OprF, 7,8 and 9 with OprE, 10 with A-type flagellin, 20 with Opr86 and 21 with LPS assembly protein LptD. OprH was the protein match found for protein spot 6 seen in control and tetradecane-treated cells. The protein spots 51, 53, 71, 75 and 100 that were absent from control, but were seen in n-dodecane, n-decane, n-octane, n-heptane and n-hexane also matched with OprH on the database search.

Outer membrane proteome of *Pseudomonas aeruginosa*, has been reported to have 104 Open Reading Frames (ORFs). Functionally, these proteins belong to three major categories namely, porins, receptors and proteins with unknown function. The biggest group is that of porins consisting of 17 proteins [19]. In the present study, eight differentially expressed proteins of *Pseudomonas aeruginosa* outer membrane belong to the porin classes, OprD, E, F, H, and Opr86. Porins are pore-forming proteins which are considered important for transport, both into and out of cells. General diffusion pores formed by porins allow the diffusion of hydrophilic molecules (<600 Da) without much substrate specificity [20]. Alkanes, despite their hydrophobic character also seem to have an effect on outer membrane porins which are usually known to form channels that enable the passage of hydrophilic molecules such as phenol [8], [21], chlorophenol and antibiotics [22]. The entry of such solvents across the outer membrane into the cell, results in the leakage of protons across the outer membrane and a drop in the proton motive force across the inner bacterial membrane [24]. The change in the porin levels seen in presence of alkanes is likely to be an attempt by the cell to restore this proton imbalance.

Porins OprE, OprH, Opr86 and a lipopolysaccharide assembly protein, LptD were up-regulated in presence of solvents. OprE is an anaerobically induced outer membrane porin [38] predicted to take up either arginine or proline [39]. Its up-regulation in the present study can be explained by the distinct anaerobic environment created by the high concentration, 33% (v/v), of organic solvents used for growing the bacterial cells. This indicates that cells grown in solvent, face low oxygen and anaerobic like environment. OprH is up-regulated in all except n-tetradecane-treated cells, where it is infact down-regulated. OprH expression has been shown to result in the blocking of self-promoted uptake of antimicrobials [25]. The up-regulation of OprH in the present case indicates enhanced uptake of solvents into cell. Opr86 is responsible for transport of small molecules, cell viability and outer membrane protein assembly. When Opr86 is depleted, visible changes are evident in the morphology of the cell [26], [27]. The up-regulation of Opr86 in the present case may be due to the damage to the outer membrane caused by the solvents and an imminent need thereof, for its repair. In our previous studies related to the solvent effect on the cells of *P. aeruginosa*, we have shown the damage caused to the membrane in presence of toxic solvents and also the intracellular accumulation of solvents [13]. Hence, the up-regulation of OprH in presence of organic solvents of lower log P_ow_ value and of Opr86 in all the solvent-treated samples, supports our previous observation. LptD along with LptE is an essential outer membrane protein involved with lipopolysaccharide assembly in the outer membrane as part of the outer membrane LPS assembly [28], [29]. The up-regulation of this protein in presence of alkanes is likely to be another measure in the direction of restoration of normal structure of the cell.

The OprD, OprF, and A-type flagellin are down-regulated in response to all the alkanes. Their respective functions are that (i) OprD, as a component of a multi-drug resistance efflux pump, has been implicated in carbapenem resistance. It also acts as a specific channel for basic aminoacids and small peptides [23], [30] and possibly alkanes in the present case (ii) OprF is the major non specific porin of *Pseudomonas aeruginosa* that is known to form large outer membrane channels that permit the passage of various solutes, albeit slowly [31]. Thus, the down-regulation of these porins appropriately explains the adaption of cells to block the solvent passage.

Besides porins, A-type flagellin, shows a significant decrease in expression across all the solvent treated cells. We hypothesize that this may be due to a need for the bacterial cells to reduce their permeable surface area. This is a new finding that has not been reported so far, in bacteria. However, studies on the algal flagellate, *Chlamydomonas* have shown that high concentrations of chemical agents in the cellular environment of the algal cells, caused the flagella to be shed. This enables the cells to reduce their permeable surface area [32]. This forms the highlight of this work.

### Protein hydrophobicity

The subcellular localization of the proteins visualized on the 2-D gels was further confirmed by determination of their GRAVY values using the online GRAVY calculator (www.gravy-calculator.de). All the detected proteins had a negative GRAVY ranging from −0.13 to −0.63 indicating their hydrophilic nature and localization to the outer membrane protein.

The present study describes the changes in outer membrane proteome of a known solvent-tolerant *P. aeruginosa* cells as a result of solvent exposure. Briefly, the results show that seventeen proteins were differentially expressed when grown in presence of alkanes. These differentially expressed proteins were identified by LC-ESI MS/MS. Their identity matched with porins OprD, OprE, OprF, OprH, Opr86, LPS assembly protein and A-type flagellin. The results showed significant down-regulation of the flagellin A protein, OprF, OprD; up-regulation and modification of OprH, OprE, Opr86 and Lpt D in presence of organic solvents. These changes reflect upon the adaptation of the bacterial cells for survival under solvent-rich conditions in which the porins play an important role.
